# Enhanced reversal of ABCG2‐mediated drug resistance by replacing a phenyl ring in baicalein with a *meta*‐carborane

**DOI:** 10.1002/1878-0261.13527

**Published:** 2023-10-05

**Authors:** Lydia Kuhnert, Robert Kuhnert, Menyhárt B. Sárosi, Cathleen Lakoma, Birte K. Scholz, Peter Lönnecke, Evamarie Hey‐Hawkins, Walther Honscha

**Affiliations:** ^1^ Faculty of Veterinary Medicine, Institute of Pharmacology, Pharmacy and Toxicology Universität Leipzig Germany; ^2^ Faculty of Chemistry and Mineralogy, Institute of Inorganic Chemistry Universität Leipzig Germany; ^3^ Center for Nanosystems Chemistry (CNC) Universität Würzburg Germany; ^4^ Institut für Organische Chemie Universität Würzburg Germany

**Keywords:** ABCG2, baicalein, breast cancer resistance protein/BCRP, carborane, drug resistance

## Abstract

Success of chemotherapy is often hampered by multidrug resistance. One mechanism for drug resistance is the elimination of anticancer drugs through drug transporters, such as breast cancer resistance protein (BCRP; also known as ABCG2), and causes a poor 5‐year survival rate of human patients. Co‐treatment of chemotherapeutics and natural compounds, such as baicalein, is used to prevent chemotherapeutic resistance but is limited by rapid metabolism. Boron‐based clusters as *meta*‐carborane are very promising phenyl mimetics to increase target affinity; we therefore investigated the replacement of a phenyl ring in baicalein by a *meta*‐carborane to improve its affinity towards the human ABCG2 efflux transporter. Baicalein strongly inhibited the ABCG2‐mediated efflux and caused a fivefold increase in mitoxantrone cytotoxicity. Whereas the baicalein derivative 5,6,7‐trimethoxyflavone inhibited ABCG2 efflux activity in a concentration of 5 μm without reversing mitoxantrone resistance, its carborane analogue 5,6,7‐trimethoxyborcalein significantly enhanced the inhibitory effects in nanomolar ranges (0.1 μm) and caused a stronger increase in mitoxantrone toxicity reaching similar values as Ko143, a potent ABCG2 inhibitor. Overall, *in silico* docking and *in vitro* studies demonstrated that the modification of baicalein with *meta*‐carborane and three methoxy substituents leads to an enhanced reversal of ABCG2‐mediated drug resistance. Thus, this seems to be a promising basis for the development of efficient ABCG2 inhibitors.

## Graphical abstract

Multidrug resistance mediated by the breast cancer resistance protein (BCRP, ABCG2) is challenging in cancer therapy. Therefore, combined use of ABCG2 inhibitors with anticancer agents seems to be useful to overcome drug resistance. Based on baicalein, used in traditional Chinese medicine, we replaced a phenyl group by boran‐based cluster (*meta*‐carborane) to enhance ABCG2 inhibition and successfully reversed mitoxantrone resistance.

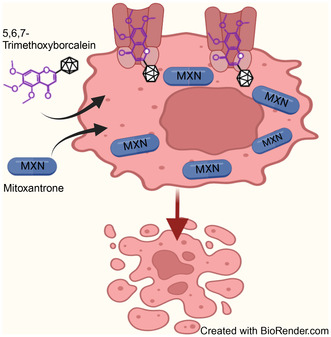

**Abbreviations**
ABCG2: ATP‐binding cassette transporter subfamily G2
BCA: bicinchoninic acid assay
BCRP: breast cancer resistance protein
carborane: dicarba‐*closo*‐dodecaborane
DMSO: dimethyl sulfoxide
hABCG2: human ABCG2
IC_50_
: inhibitory concentration
LGA: Lamarckian genetic algorithm
MDCKII: Madin‐Darby canine kidney
MEM: minimum essential medium
MXN: mitoxantrone
PDB ID: protein data bank identification number
RFU: relative fluorescence unit
SDS: sodium dodecyl sulfonate
WST‐1: water‐soluble tetrazolium 1

## 1 Introduction

For decades, the improvement of cancer treatment by new anticancer drugs is a broad field of research. Particularly, chemotherapeutic resistance leads to poor prognoses, and huge efforts were made to improve drug therapies [1]. Tumour cells exhibit multidrug resistance by various mechanisms; one of them is the expression of efflux transporters [2]. The human ATP‐binding cassette transporter subgroup G2 (ABCG2, also named BCRP) is expressed in different cancer cells and eliminates a broad spectrum of drugs, like doxorubicin, mitoxantrone and several tyrosine kinase inhibitors, from the cells by ATP‐depending efflux. This leads to multidrug resistance by reducing intracellular drug concentration to subtherapeutic levels and affects the success of chemotherapy [3]. In addition, for melanoma and colon cancer a negative correlation between ABCG2 expression and poor 5‐year prognosis is reported [4]. Novel therapeutic approaches in human medicine focus on concurrent application of anticancer agents and efflux membrane inhibitors to reverse chemoresistance, even by using natural herbs [1]. At present, no specific ABCG2 inhibitors have been successfully passed the clinical trials and are available for clinical use [3].

As confirmed by several studies, the herbal ingredient of *Scutellaria baicalensis* baicalein is used in traditional Chinese medicine in regard to its anticancer effects against breast cancer cells, hepatocellular carcinoma, leukaemia, colon and prostate cancer cells by affecting different pathways [1, 5, 6, 7]. As described in the literature, baicalein also inhibits the human ABCG2 (hABCG2) efflux activity in RPMI8226 cells [8]. The rapid metabolism and recycling of natural flavones *in vivo* complicates the prediction of the pharmacokinetic properties [9]. Hence, metabolically stable boron clusters, known as carboranes (carbaboranes, dicarba‐*closo*‐dodecaboranes) as substituents instead of phenyl groups, were used to prevent enzyme degradation during biotransformation [10, 11, 12]. Moreover, the exchange of phenyl groups by carboranes is described to increase the target affinity, for example, of carborane‐based cyclooxygenase inhibitors [12]. Furthermore, carboranes have various beneficial features: their high hydrophobicity improves the access through cellular membranes, and due to their similar diameter, bioisosteric replacement of phenyl rings in pharmacologically active substances by carboranes is a useful approach [12, 13]. Particularly, several studies verified that the substitution of flavones with methoxy groups increases the affinity towards the hABCG2‐binding pocket [14, 15]. Thus, we systematically analysed the modification of baicalein with carboranes and methoxy groups to provide a new approach to overcome drug resistance.

## 2 Materials and methods

### 2.1 Synthesis

The synthesis of the carborane derivatives, borcalein and 5,6,7‐trimethoxyborcalein was performed as described previously [16], and the synthesis of 5,6,7‐trimethoxy‐4*H*‐chromen‐4‐one was performed according to the literature [17]. 5,6,7‐Trimethoxyflavone and baicalein are commercially available.

### 2.2 Biological studies

#### 2.2.1 Materials and methods

Water‐soluble tetrazolium 1 (WST‐1) was obtained from MERCK KGaA (Darmstadt, Germany), Dulbecco's PBS from Biowest SAS (Nuaillé, France) and sodium dodecyl sulfonate and DMSO (dimethyl sulfoxide) from Carl Roth (Karlsruhe, Germany). All other reagents were purchased from Sigma (St. Louis, MO, USA) and Sigma‐Aldrich (Munich, Germany).

#### 2.2.2 Cultivation of the cells

Madin‐Darby canine kidney cells (MDCKII, RRID: CVCL_0424) and their related cells stably transfected with hABCG2 (MDCKII‐hABCG2) were purchased from Alfred Schinkel (Het Nederlands Kanker Instituut, Amsterdam, Netherlands) [18] and were cultivated in MEM (minimum essential medium) with Earle's Salts (2.2 g·L^−1^ NaHCO_3_, stable glutamine; Biochrom, Berlin, Germany) supplemented with 10% (*v/v*) fetal calf serum (Life Technology, Karlsruhe, Germany), 1% (*v/v*) nonessential amino acids (Biochrom, Berlin, Germany), 100 U·mL^−1^ penicillin and 100 μg·mL^−1^ streptomycin (Biochrom, Berlin, Germany). Cells were grown in humidified atmosphere (37 °C, 5% pCO_2_) and were subcultured every 3–4 days using 0.05% trypsin/0.02% EDTA (Biochrom, Berlin, Germany).

#### 2.2.3 Proof of absence of mycoplasma and identity of the cells

Due to MDCKII cells originated from dogs, no genomic sequence is available for cell authentication. To identify the cells and to proof the absence of cross‐contamination, mRNA expression of the foreign gene hABCG2 was routinely investigated by PCR. MDCKII and MDCKII‐hABCG2 cells with an amount of 3.5 × 10^4^ cells were collected in RNA*later* (Thermo Fisher Scientific Baltics UAB, Vilnius, Lithuania). RNA isolation, cDNA transcription and PCR were performed using RNAeasy Plus Mini Kit (Qiagen, Hilden, Germany), ReversedAid First Strand cDNA Synthesis Kit (Thermo Fisher Scientific Baltics UAB, Vilnius, Lithuania) or DreamTaq Green PCR Master Mix (Thermo Fisher Scientific Baltics UAB, Vilnius, Lithuania) according to the manufacturer's instructions. Specific primer for hABCG2 (forward primer: 5′‐gctgaattacatcaactttccgggggtga‐3′; reverse primer: 5′‐ggattgtttcctgttgcattgagtcctgg‐3′) were obtained from Biomers (Ulm, Germany) and used for PCR. PCR was carried out by an initial denaturation step (95 °C, 2 min) followed by 35 cycles of annealing (95 °C, 30 s), amplification (66 °C, 30 s) and extension (72 °C, 45 s). A final elongation period at 72 °C for 5 min was examined. The PCR products were analysed by agarose gel electrophoresis. Moreover, the functional hABCG2 activity using Hoechst 33342 accumulation assay is described in Section 2.2.5. Non‐transfected MDCKII cells showed higher accumulation of Hoechst 33342 dye than MDCKII‐hABCG2 cells and are not affected by the applied compounds. All experiments were performed with mycoplasma‐free cells. To validate the absence of mycoplasma, cell supernatants were collected from cells cultured for 3–4 days and PCR with specific primers (Biomers, Ulm, Germany) to detect mycoplasma 16S rRNA gene was used according to the standard national guideline (§ 28 GenTG [19]). After DNA extraction, PCR was executed by an initial denaturation step of 2 min at 95 °C followed by 35 cycles of annealing (95 °C, 30 s), amplification (57 °C, 10 s) and extension (72 °C, 45 s). A final elongation step (72 °C, 5 min) was added. Subsequently, agarose gel electrophoresis analysis was performed in comparison with the positive control.

#### 2.2.4 Determination of cell viability by WST‐1 assay

MDCKII‐hABCG2 cells (2 × 10^4^ cells·mL^−1^) and their parental MDCKII cells (3 × 10^4^ cells·mL^−1^) were seeded in 96‐well plates (200 μL per well; TPP, Trasadingen, Switzerland). After 48 h, cells were incubated with increasing concentrations up to 50 μm of selected compounds with the exception of Ko143 (up to 10 μm). Cells treated with 0.1% Triton X‐100 served as positive control, and untreated cells were used as negative control. The treatment was renewed once a day. After 48 h incubation, the substance‐specific cytotoxicity was determined by WST‐1 assay as described previously [20]. The cell viability is reflected by an increase in formazan formation and was measured after 1 h at 450 nm by a microplate reader (Tecan Sunrise, Crailsheim, Germany).

#### 2.2.5 Determination of ABCG2 interaction with Hoechst 33342 accumulation assay

Hoechst 33342 accumulation assay was used to detect an interaction of the investigated compounds with the hABCG2 transporter as described previously [20]. Two hundred μL per well of MDCKII‐hABCG2 (2 × 10^4^ cells·mL^−1^) and MDCKII (3 × 10^4^ cells·mL^−1^) cells was seeded into 96‐well plates and cultured for 72 h. Afterwards, subconfluent monolayers were treated with increasing concentrations (0.1, 0.5, 1 and 5 μm) of selected compounds, 5 μm Ko143 or 0.1% DMSO as solvent control for 4 h. The intracellular amount of Hoechst 33342 was detected by spectrofluorometry (360 nm excitation/465 nm emission wavelengths; Tecan Infinite F200 Pro, Crailsheim, Germany) and relative fluorescence units (RFU) were correlated to the protein amount quantified by bicinchoninic acid assay (BCA, Thermo Scientific, Rockford, USA) following the manufacturer's instructions.

#### 2.2.6 Determination of autofluorescence

After seeding and treatment of the MDCKII‐hABCG2 and MDCKII cells as described for Hoechst accumulation assay (Section 2.2.5), autofluorescence was investigated as described previously [20]. The intracellular fluorescence was measured by spectrofluorometer (360 nm excitation/465 nm emission wavelengths, Tecan Infinite F200 Pro, Crailsheim, Germany). After the background of non‐treated cells was subtracted from the obtained intracellular fluorescence, total intracellular fluorescence was correlated to the protein amount quantified by BCA assay. An autofluorescence was defined as a significant increase in total intracellular fluorescence unit (RFU) in comparison with solvent‐treated control.

#### 2.2.7 Determination of reversal of mitoxantrone resistance by WST‐1 assay

MDCKII‐hABCG2 and MDCKII cells were seeded in 96‐well plates as described in Section 2.2.4 (TPP, Trasadingen, Switzerland). Cells were incubated with increasing concentrations up to 50 μm of mitoxantrone (MXN) alone or in combination with selected compounds (1 μm and/or 5 μm) for 48 h. Cells treated with 0.1% Triton X‐100 served as positive control, and untreated MDCKII‐hABCG2 or MDCKII cells were used as negative control. The treatment was renewed once a day. Subsequently, the substance‐specific cytotoxicity was determined by WST‐1 assay as described previously [20]. IC_50_ values, defined as 50% reduced cell viability, were calculated. The left shift factor was calculated by dividing IC_50_ MXN alone by IC_50_ MXN in combination with the selected compound as shown for Ko143 (1 μm) in the formula:

$$\text{Left shit}\;{\text{factor}}_{1\;\mu \text{ᴍ}\;\mathrm{Ko}143}=\frac{\left({\mathrm{IC}}_{50\;\mathrm{MXN}}-{\mathrm{SEM}}_{\mathrm{MXN}}\right)}{\left({\mathrm{IC}}_{50\;\mathrm{MXN}+1\;\mu \text{ᴍ}\;\mathrm{Ko}143}+{\mathrm{SEM}}_{\mathrm{MXN}+1\;\mu \text{ᴍ}\;\mathrm{Ko}143}\right)}$$

### 2.3 Molecular docking

Ligand structures were constructed with avogadro 1.2 [21]. Ligand geometries were optimized with ORCA [22] using the PBEh‐3c method [23]. The atomic charges for each ligand were derived from the RESP procedure [24, 25, 26] with Gaussian 09 (HF/6–31 + G**) and the *antechamber* programme of the AmberTools 17 package [27]. The crystal structure of human multidrug transporter ABCG2 [28] was obtained from the Protein Data Bank (www.rcsb.org) [29]. Ligands, all nonstandard residues, and all water molecules were removed from PDB ID: 5NJ3 the crystal structure with the UCSF chimera package [30]. The crystal structure was prepared for docking with autodock Tools 1.5.6 [31]. The macromolecule and ligand structures were prepared for docking according to a previously reported protocol [32]. A new atom type (B) was defined for the boron atoms containing the force field parameters available for docking of carborane‐containing ligands [33]. The docking area was defined using autogrid 4.2.5. [34]. A 108 × 66 × 86 Å^3^ three‐dimensional affinity grid was placed around the drug‐binding pocket of the ABCG2 crystal structure (PDB ID: 5NJ3) [28]. A 0.375 Å grid point spacing was used throughout. Docking was performed with autodock 4.2.5.1 [31] according to a previously reported protocol [32]. Details are given here for completeness: The Lamarckian genetic algorithm (LGA) was selected for the ligand conformational search [34]; parameters used for LGA: population size of 150 individuals; 30 × 10^6^ energy; maximum of 27 000 generations; one top individual to survive to the next generation automatically; mutation rate of 0.02; crossover rate of 0.8; 100 docking runs; random initial positions and conformations; the probability of performing a local search on an individual in the population was set to 0.06, and the maximum number of iterations per local search was set to 300. The final docked conformations were grouped using a tolerance of 1.5 Å root‐mean‐square deviations. The setup of the docking protocols and the analysis of the results were done with autodock Tools 1.5.6. [31]. The standard error of the AutoDock free energy scoring function was 2–3 kcal·mol^−1^ [31]. All figures were rendered with the UCSF chimera package [30].

### 2.4 Statistical analyses

Statistical analyses and calculation of IC_50_ (inhibitory concentration) values were performed with sigmaplot 14.5 (Systec Software, San Jose, CA, USA). All data were tested for normality by Shapiro–Wilk. All IC_50_ values were calculated from at least three independent experiments by nonlinear regression with four‐parametric logistic curve analyses. Substance‐specific IC_50_ values are defined as substance‐specific concentrations decreasing cell viability to 50%.

## 3 Results and Discussion

### 3.1 Synthesis

**Fig. 1:**
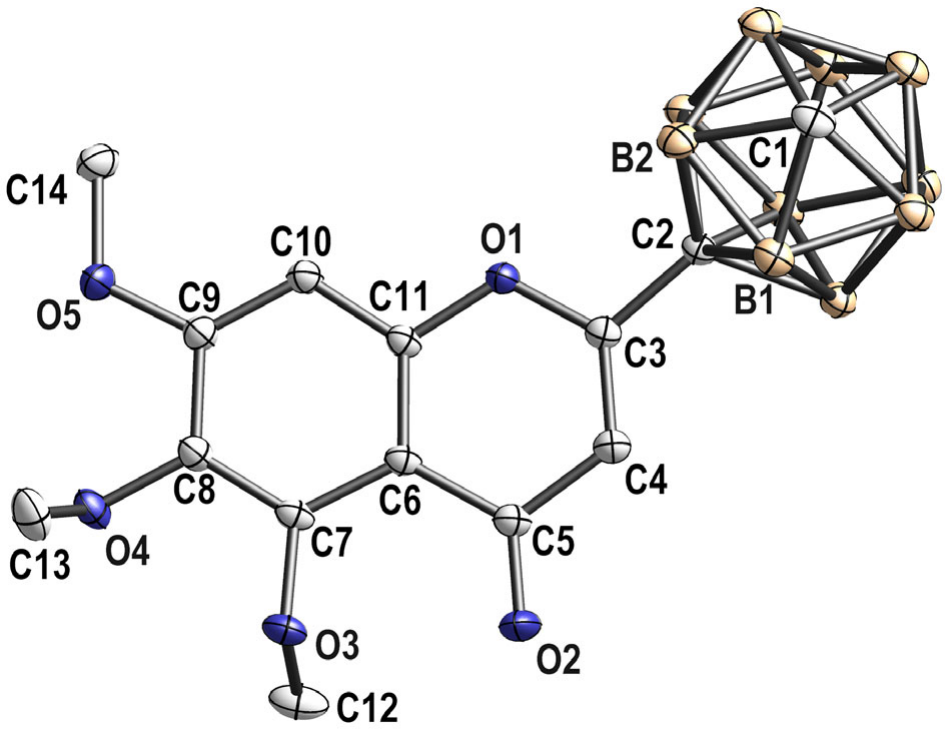
Molecular structure of 5,6,7‐trimethoxyborcalein. Selected bond lengths (Å) and angles (°): C(1)–B(1) 1.688(1), C(1)–B(2) 1.693(1), C(2)–B(1) 1.705(1), C(2)–B(2) 1.710(1), C(2)–C(3) 1.496(1), O(1)–C(3) 1.355(9), C(3)–C(4) 1.339(1), C(4)–C(5) 1.451(1), O(2)–C(5) 1.231(9), O(3)–C(7) 1.368(9), O(3)–C(12) 1.431(1), C(1)–B(2)–C(2) 100.97(6), C(3)–C(2)–B(2) 116.46(6), O(1)–C(3)–C(2) 111.23(6), C(3)–O(1)–C(11) 118.93(6), C(4)–C(3)–C(2) 125.32(7), O(3)–C(7)–C(6) 120.89(7) and C(7)–O(3)–C(12) 113.53(7). Figure was generated with Diamond (Klaus Brandenburg, Diamond 4, version 4.6.8, Crystal Impact GbR, Bonn, Germany). Ellipsoids are shown at 50% probability, and hydrogen atoms are omitted for clarity (for further details see Table S1).

In traditional Chinese medicine, baicalein is used to treat different types of cancer [1, 5, 6, 7] and combined application with anticancer drugs may enhance chemotherapy through ABCG2 inhibition. Due to the rapid metabolism of baicalein, the replacement of phenyl ring by carborane is used to improve metabolic stability and affinity towards ABCG2‐binding pocket. Furthermore, substitution of flavones with methoxy groups is described as increasing ABCG2 inhibition [14, 20]; hence, derivatives substituted with three methoxy groups were further investigated. We have synthesized compounds **3** (5,6,7‐trimethoxyborcalein) and **4** (borcalein) as baicalein derivatives as described previously [16]. 5,6,7‐Trimethoxyborcalein was fully characterized; the structure was elucidated by heteronuclear multiple bond correlation nuclear magnetic resonance spectroscopy and X‐ray crystallography (Fig. 1 and Table S1). To evaluate the replacement of a phenyl ring by carborane and the impact on ABCG2 inhibition, baicalein, 5,6,7‐trimethoxyflavone and 5,6,7‐trimethoxy‐4*H*‐chromen‐4‐one were included in this study for comparison with borcalein and 5,6,7‐trimethoxyborcalein, respectively.

### 3.2 Cytotoxicity to MDCKII cells

Water‐soluble tetrazolium 1 assay was performed to exclude cytotoxic effects of the selected compounds in the further experiments. MDCKII cells expressing the hABCG2 transporter (MDCKII‐hABCG2) and their parental cells (MDCKII) were treated with the investigated compounds for 48 h. Afterwards, the substance‐specific cytotoxicity was determined by WST‐1 assay. The highest stock solution of investigated compounds was 50 mm (Ko143 10 mm) to achieve a solvent concentration of 0.1% DMSO. Due to the limited solubility, higher concentrations could not be examined. Ko143 and solvent (0.1% DMSO) caused no cytotoxicity towards MDCKII cells up to 50 μm (Fig. S1A). While phenyl‐containing baicalein (Fig. S2A) caused no cytotoxicity, for its carborane analogue borcalein an IC_50_ value for MDCKII‐hABCG2 of 34.3 μm and for MDCKII of 12.6 μm was determined (Fig. S2B). No cytotoxicity towards MDCKII cells was observed for treatment with 5,6,7‐trimethoxyflavone (Fig. S2C). A treatment with its carborane analogue 5,6,7‐trimethoxyborcalein caused a reduced cell viability in MDCKII‐hABCG2 cells (50 μm) and MDCKII (10 μm and 50 μm, Fig. S2D). These subtoxic effects did not affect the following experiments due to the concentrations used up to 5 μm. For the basic compound without phenyl or carborane substitution, 5,6,7‐trimethoxychromen‐4*H*‐one, a decreased viability of MDCKII‐hABCG2 cells of about 20% was detected (Fig. S2E). Overall, the carborane‐based compounds seemed to be more toxic towards MDCKII cells than their phenyl analogues. With exception of borcalein, no significant impact on cell viability below 5 μm was detected, so further experiments up to 5 μm could be investigated. An increased toxicity of borcalein (Fig. S2C) was also observed in different cancer cell lines and underlines an unspecific toxic behaviour [16]. Thus, borcalein was only measured up to 1 μm in MDCKII‐hABCG2 and MDCKII cells in ABCG2 inhibition studies.

### 3.3 ABCG2 inhibition in MDCKII cells

**Fig. 2:**
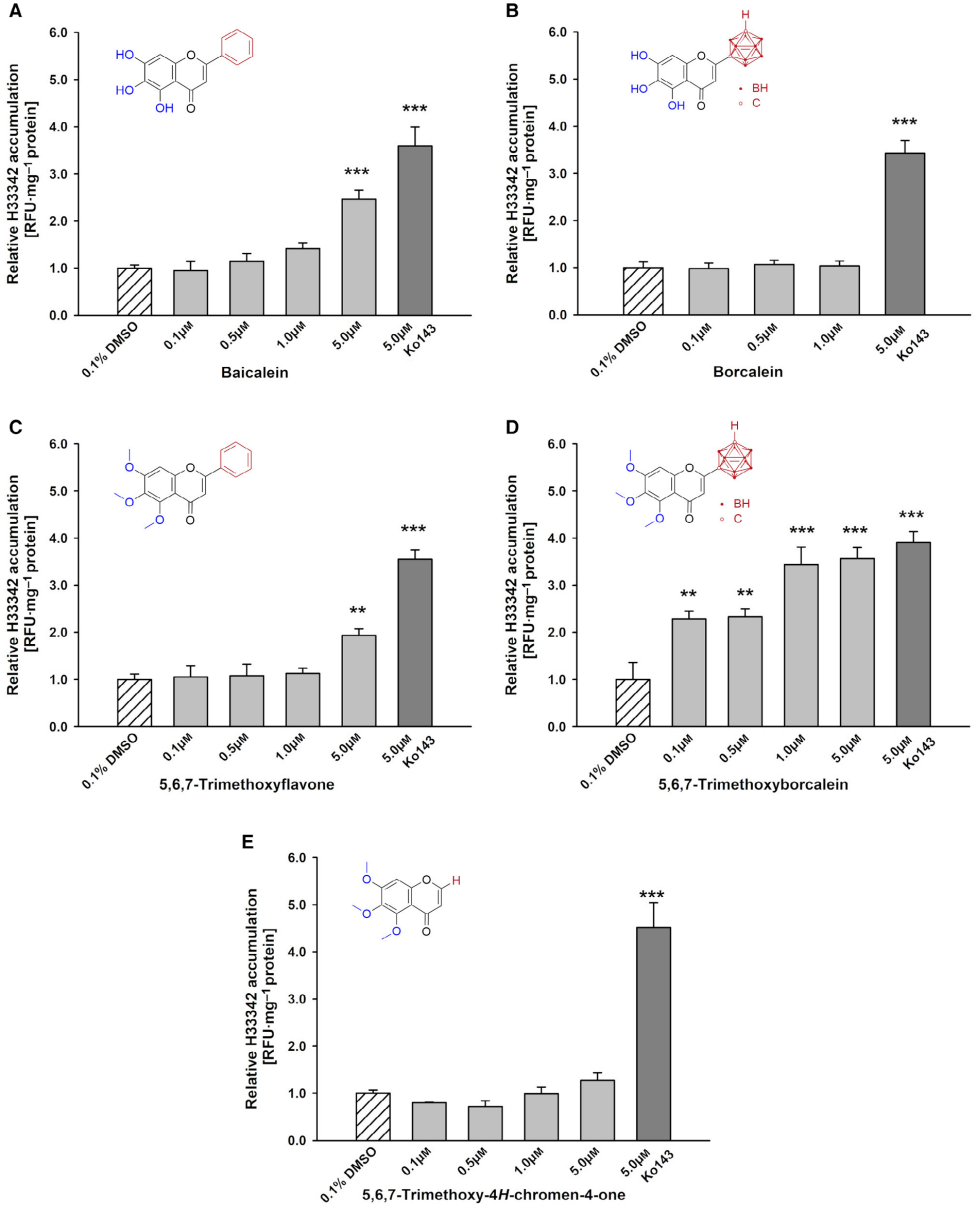
Inhibition of hABCG2 in MDCKII cells. Relative Hoechst 33342 accumulation in MDCKII‐hABCG2 and MDCKII cells after treatment with (A) baicalein, (B) borcalein, (C) 5,6,7‐trimethoxyflavone, (D) 5,6,7‐trimethoxyborcalein and (E) 5,6,7‐trimethoxy‐4*H*‐chromen‐4‐one. Data were normalized to their solvent control (0.1% DMSO) set as 1 (mean ± SEM, *N* = 4, one‐way ANOVA with Holm‐Šidák *post hoc* test, * significant difference in comparison with the control: ***P ≤ 0.001, **P ≤ 0.01).

The Hoechst 33342 accumulation assay is a well‐established tool to detect an ABCG2 inhibition. An inhibition of the ABCG2 efflux transporter is reflected by an intracellular increase in the Hoechst 33342 dye in comparison with the solvent control (0.1% DMSO). To exclude the influence of other membrane transporters in this study, the relation of Hoechst accumulation in MDCKII‐hABCG2 to parental MDCKII cells was determined. No changes in intracellular accumulation in MDCKII cells were observed. The known ABCG2 inhibitor Ko143 caused a significant increase in the fluorescent dye in 0.1 μm (3‐fold) up to 5 μm (3.5‐fold; Fig. S1B) and was used as positive control. While 5 μm of the parental compound baicalein led to a 2.5‐fold significantly increased amount of Hoechst 33342 dye (Fig. 2A), the carborane analogue, borcalein, did not (Fig. 2B). Therefore, baicalein is able to inhibit the hABCG2 efflux activity as described in the literature [8].

Due to methoxy‐substituted flavones being known to enhance affinity towards ABCG2 [14, 15], methoxy‐derivatives of baicalein (5,6,7‐trimethoxyflavone) and borcalein (5,6,7‐trimethoxyborcalein) were further investigated. Additionally, the basic compound 5,6,7‐trimethoxy‐4*H*‐chromen‐4‐one was added to this study to compare the impact of the boron‐based cluster with phenyl or proton substitution.

Cells treated with 5 μm 5,6,7‐trimethoxyflavone, the trimethoxy derivative of baicalein, significantly doubled the Hoechst 33342 accumulation (Fig. 2C), and all applied concentrations (0.1 μm up to 5 μm) of carborane‐based analogue 5,6,7‐trimethoxyborcalein enhanced the intracellular dye amount by two to four times, similar to the positive control Ko143 (Fig. 2D). Interestingly, no increased dye accumulation was detected for 5,6,7‐trimethoxy‐4*H*‐chromen‐4‐one containing neither phenyl ring nor *meta*‐carborane cluster (Fig. 2E). Thus, a combination of *meta*‐carborane and three methoxy groups is crucial to enhance ABCG2 affinity.

### 3.4 Absence of autofluorescence

Natural fluorescence of the investigated compounds may disturb the Hoechst accumulation assay. Therefore, cells were treated with the compounds for 4 h and intracellular autofluorescence of the compounds was determined. As shown in Figs S3–S5, no autofluorescence was detectable either in MDCKII or in MDCKII‐hABCG2 cells, which may influence the obtained results.

### 3.5 Molecular docking studies

**Fig. 3:**
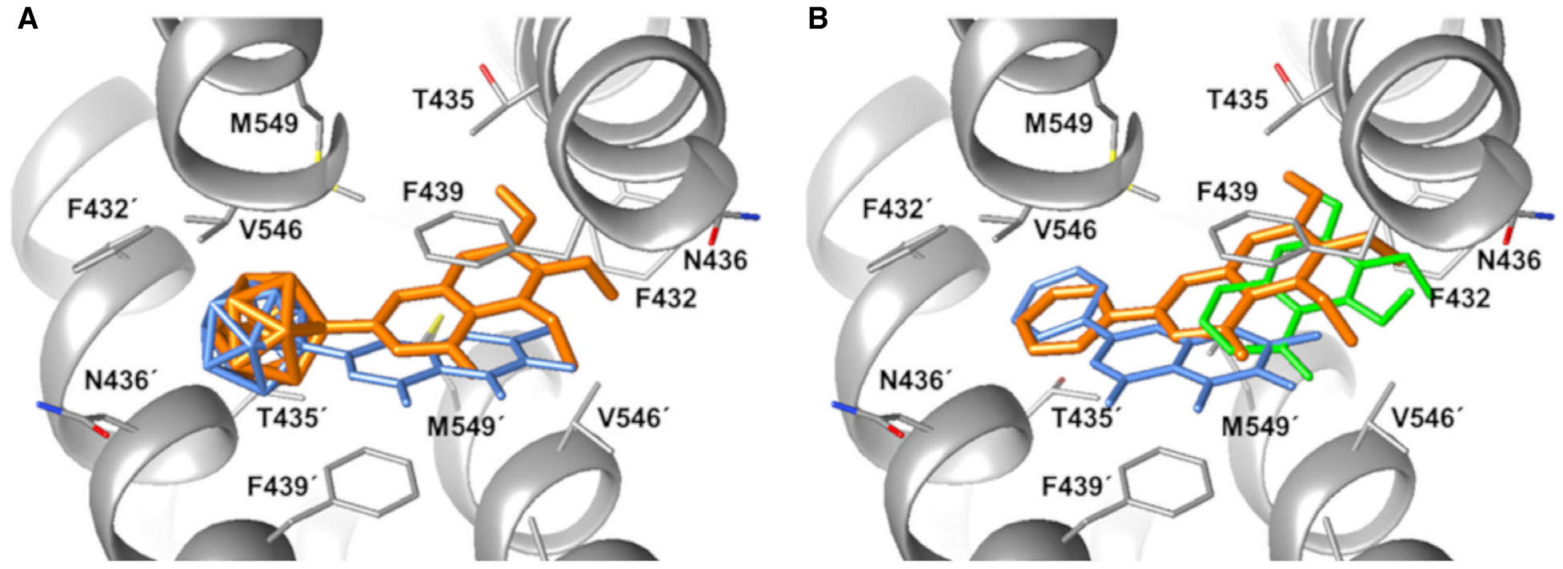
Docking into the cavity 1 of the ABCG2 crystal structure (PDB ID: 5NJ3). (A) Docking of carboranyl derivatives 5,6,7‐trimethoxyborcalein (orange) and borcalein (blue) with their predicted binding free energies of −5.0, and −2.0 kcal·mol^−1^, respectively. (B) Docking of baicalein (blue), 5,6,7‐trimethoxyflavone (orange) and of 5,6,7‐trimethoxy‐4*H*‐chromen‐4‐one (green) and their binding free energies of −2.2, −5.7 and −2.8 kcal·mol^−1^, respectively. The corresponding residues of the two ABCG2 monomers are labelled accordingly. Figures were created as described in the Materials and Methods: Section 2.3. Hydrogen atoms are not shown for clarity.

For a deeper understanding of the interaction with ABCG2, *in silico* molecular docking studies were performed [3]. The recently published crystal structure of hABCG2 (PDB ID: 5NJ3) [28] was used for docking the synthesized compounds (Fig. 3), and the binding energies towards avity 1 of hABCG2 protein were determined (Table S2). The calculation of the highest binding free energy towards hABCG2 is used to identify the ABCG2 inhibitor with the highest affinity and to describe compound−protein interactions [3]. The docking studies predicted similar orientations for borcalein and 5,6,7‐trimethoxyborcalein (Fig. 3) within cavity 1 of ABCG2. Overall, the studies favoured the binding of 5,6,7‐trimethoxyborcalein (Table S2) and showed an interaction of *meta*‐carborane with hydrophobic residues of the ABCG2‐binding pocket (Fig. 3A). Baicalein (Fig. 3B) and borcalein (Fig. 3A) exhibit similar binding free energies about −2.0 kcal·mol^−1^, but as shown by the *in vitro* results, borcalein caused unspecific toxicity in low concentration ranges. From all examined compounds, the highest binding free energy was determined for 5,6,7‐trimethoxyborcalein (−5.0 kcal·mol^−1^) and its phenyl analogue 5,6,7‐trimethoxyflavone (−5.7 kcal·mol^−1^). The hABCG2 protein is formed by two symmetric monomers; accordingly, molecule binding can be symmetric or asymmetric. While symmetric inhibition is reflected by two bound molecules in cavity 1 (one molecule bound to one monomer of ABCG2), a binding of only one molecule to both monomers simultaneously is defined as asymmetric. Only one molecule was predicted to bind into the central ABCG2 cavity formed by the two monomers reflecting an asymmetric inhibition mode. This agrees with the crystal structure of ABCG2 complexed with compound MB136 [35]. Only one molecule of MB136 was found to bind in an asymmetric fashion to ABCG2 causing a maximal inhibition at a molar ratio of 1 : 1. However, the presence of multiple modes of binding could not be excluded. Jackson et al. [35] also speculated that inhibitors remain tightly bound in cavity 1 of ABCG2, blocking access and preventing conformational changes required for substrate transport. In accordance with our *in vitro* results and the literature [3], substitution with three methoxy groups enhanced the affinity towards hABCG2. As shown, the high affinity of 5,6,7‐trimethoxyflavones in combination with the steric demand of a three‐dimensional *meta*‐carborane seems to be crucial for enhanced ABCG2 inhibition.

### 3.6 Reversal of multidrug resistance

**Fig. 4:**
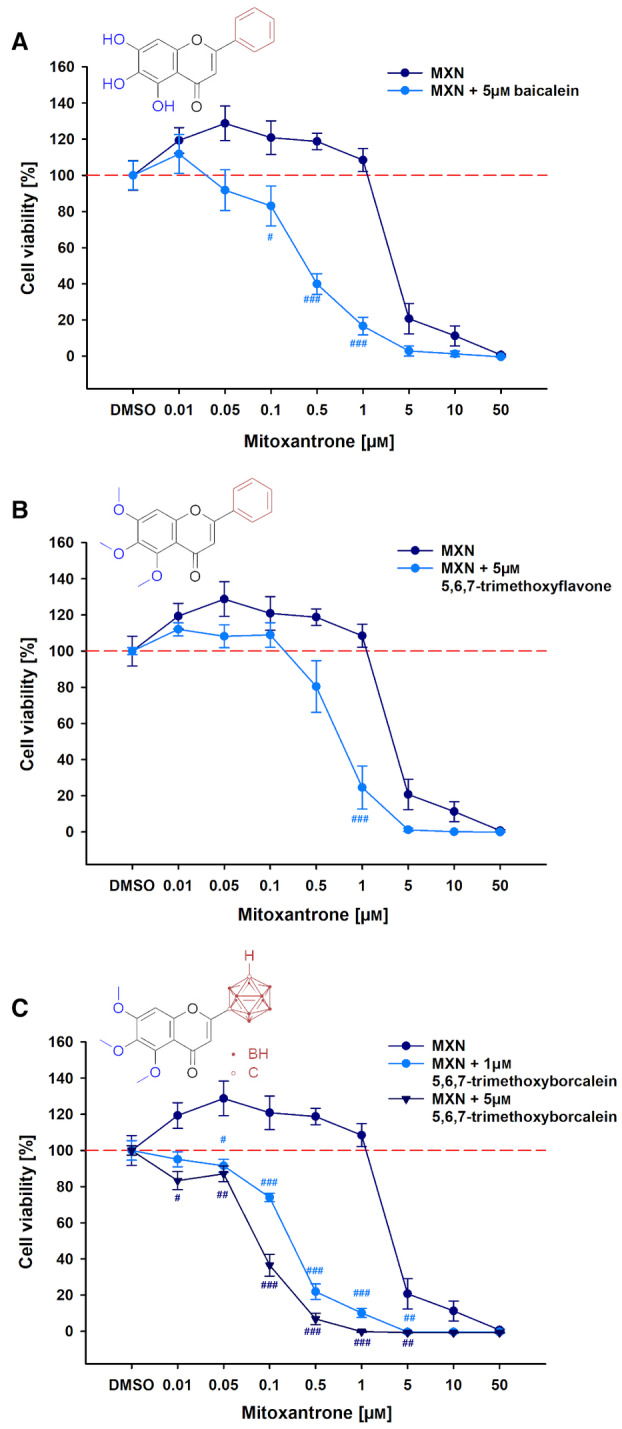
Reversal of ABCG2‐mediated mitoxantrone resistance. MDCKII cells were treated with increasing concentrations of mitoxantrone with or without (A) baicalein (5 μm), (B) 5,6,7‐trimethoxyflavone (5 μm) and (C) 5,6,7‐trimethoxyborcalein (1 μm, 5 μm) for 48 h. Afterwards, cell viability was measured by WST‐1. Data are shown as mean SEM (*N* = 3, *n* = 9, two‐way ANOVA with Holm‐Šidák *post hoc* test, ^#^ significant difference in comparison with MXN treatment alone: ^###^ *P* ≤ 0.001, ^##^ *P* ≤ 0.01, ^#^ *P* ≤ 0.05).

Mitoxantrone is an approved anticancer agent to treat metastatic breast cancer, non‐Hodgkin lymphoma and acute myeloid lymphoma [36]. Tumour cells with multidrug resistance reduce intracellular amounts of MXN by ABCG2. Therefore, a co‐treatment of the investigated compounds with MXN was proven to reverse the ABCG2‐mediated multidrug resistance in MDCKII‐hABCG2 cells. As shown in Fig. S6, MXN was much more toxic towards MDCKII cells (IC_50_ 0.519 ± 0.042 μm) than MDCKII‐hABCG2 cells (IC_50_ 2.649 ± 0.594 μm) because of the ABCG2‐mediated efflux of the drug. A combined treatment of MXN in increasing concentrations (0.01 up to 50 μm) with 5 μm and 1 μm Ko143 represented a dose‐depending increase in the cytotoxicity towards ABCG2‐overexpressing cells (Fig. S1C). The increased toxicity is reflected by a left shift of the concentration curve of MXN as shown in Fig. S1C and is described by the left shift factor. For application of 5 μm Ko143, a strong 9‐fold left shift of IC_50_ values was determined (Table S3). While baicalein (Fig. 4B) lowered the IC_50_ value of MXN from 2.649 ± 0.594 μm to 0.310 ± 0.073 μm in a significant manner, 5,6,7‐trimethoxyflavone did not (Fig. 4B, Table S3). The flavone derivative 5,6,7‐trimethoxyflavone interacts with the ABCG2 transporter without abolishing MXN resistance, probably because it insufficiently displaces MXN as a substrate. Carborane‐containing derivative 5,6,7‐trimethoxyborcalein (Fig. 4C) caused a dose‐depending reduction of the cell viability achieving a left shift factor of 8.5‐fold (1 μm) and 21.4‐fold (5 μm) in comparison with MXN alone (Fig. 4C). Consequently, baicalein and 5,6,7‐trimethoxyborcalein reversed the ABCG2‐mediated multidrug resistance. Even if further investigations on cancer cells will be necessary, the carborane and methoxy substitution of baicalein leading to 5,6,7‐trimethoxyborcalein increases the affinity towards hABCG2‐binding pocket, which is reflected by an enhanced ABCG2 interaction and a strong reversal of mitoxantrone resistance in MDCKII cells.

## 4 Conclusion

Advanced‐stage tumours are often resistant to conventional chemotherapy due to upregulation of the human ABCG2 transporter, which eliminates anticancer drugs. Inhibition of this transporter can therefore result in overcoming drug resistance. Here, we showed the enhancement of ABCG2 inhibition and reversal of mitoxantrone resistance by the modified baicalein analogue, 5,6,7‐trimethoxyborcalein in MDCKII cells in comparison with baicalein alone. The bioisosteric exchange of a phenyl moiety by a carborane in baicalein increased unspecific toxicity, but the additional introduction of three methoxy groups enhanced the affinity towards ABCG2‐binding pocket and reversed mitoxantrone resistance. Due to its three‐dimensional steric demand, the carborane mediates a novel mode of ABCG2 inhibition that is not achieved with two‐dimensional phenyl rings or protons as substituents. Therefore, carborane‐based three methoxy‐substituted compounds appear as a promising point for further investigations on ABCG2 inhibitors to overcome drug resistance.

## Conflict of interest

The authors declare no conflict of interest.

## Author contributions

LK and RK contributed to conceptualization; LK, RK, MBS, CL, BKS and PL contributed to investigation; EH‐H and WH contributed to supervision, LK, RK, MBS, EH‐H and WH contributed to writing—original draft.

### Peer review

The peer review history for this article is available at https://www.webofscience.com/api/gateway/wos/peer‐review/10.1002/1878‐0261.13527.

## Supporting information


**Fig. S1.** Ko143‐mediated cytotoxicity, ABCG2 inhibition and reversal of mitoxantrone resistance.
**Fig. S2.** Cytotoxicity of selected compounds.
**Fig. S3.** Autofluorescence of selected compounds in MDCKII‐hABCG2 cells.
**Fig. S4.** Autofluorescence of selected compounds in MDCKII cells.
**Fig. S5.** Autofluorescence of Ko143.
**Fig. S6**. Cytotoxicity of mitoxantrone towards MDCKII‐hABCG2 and MDCKII cells.
**Table S1.** Crystal data of 5,6,7‐trimethoxyborcalein.
**Table S2.** Binding free energies of selected compounds towards human ABCG2 transporter in docking simulations.
**Table S3.** Detected left shift factors.

## Data Availability

The data underlying this article are available in this publication, and supplementary information will be provided by the corresponding author upon request. The CCDC deposition number given in Table S1 for 5,6,7‐trimethoxyborcalein contains the supplementary crystallographic data for this paper. The data can be obtained free of charge via https://www.ccdc.cam.ac.uk/structures/ or from the Cambridge Crystallographic Data Centre (12 Union Road, Cambridge CB2 1EZ, UK).
