# Supplementary material for: Enhanced reversal of ABCG2‐mediated drug resistance by replacing a phenyl ring in baicalein with a meta‐carborane
Source: Mol Oncol. 2023 Oct 5;18(2):280–90. doi: 10.1002/1878-0261.13527 (PMC10850795; doi:10.1002/1878-0261.13527)
Supplement: Supplementary file 1 — Fig. S1. Ko143‐mediated cytotoxicity, ABCG2 inhibition and reversal of mitoxantrone resistance. Fig. S2. Cytotoxicity of selected compounds. Fig. S3. Autofluorescence of selected compounds in MDCKII‐hABCG2 cells. Fig. S4. Autofluorescence of selected compounds in MDCKII cells. Fig. S5. Autofluorescence of Ko143. Fig. S6. Cytotoxicity of mitoxantrone towards MDCKII‐hABCG2 and MDCKII cells. Table S1. Crystal data of 5,6,7‐trimethoxyborcalein. Table S2. Binding free energies of selected compounds towards human ABCG2 transporter in docking simulations. Table S3. Detected left shift factors. [file MOL2-18-280-s001.pdf]

Supplementary Information for

Enhanced reversal of ABCG2-mediated drug resistance by replacing a phenyl ring in baicalein with a meta-carborane

Lydia Kuhnert[1*], Robert Kuhnert[2], Menyhárt B. Sárosi[3,4], Cathleen Lakoma[1], Birte K. Scholz[1], Peter Lönnecke[2], Evamarie Hey-Hawkins[2*] and Walther Honscha[1]

*corresponding authors: Lydia Kuhnert or Evamarie Hey-Hawkins
Email: lydia.kuhnert@vetmed.uni-leipzig.de or hey@uni-leipzig.de

This PDF file includes:

Figures S1 to S6
Tables S1 to S3
SI References

1 Figures

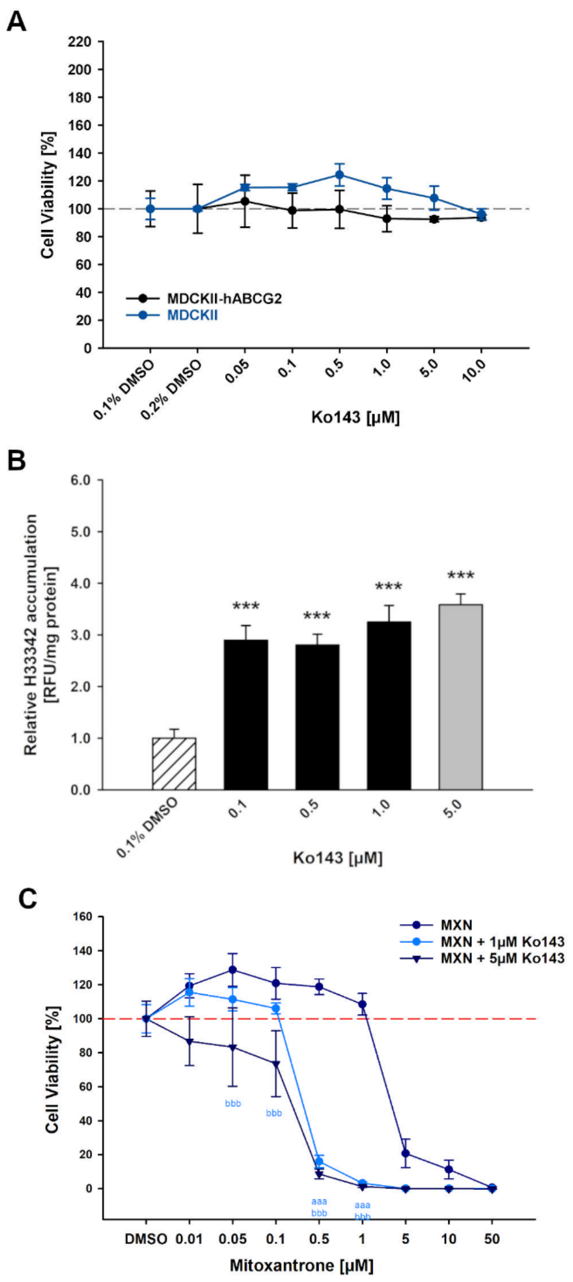

Fig. S1. Ko143-mediated cytotoxicity, ABCG2 inhibition, and reversal of mitoxantrone resistance. Impact of positive control Ko143 treatment on (A) cytotoxicity of MDCKII and MDCKII-hABCG2 cells, (B) inhibition of human ABCG2 transporter represented by relative Hoechst 33342 accumulation in MDCKII-hABCG2 to MDCKII cells, and (C) reversal of mitoxantrone (MXN) resistance in MDCKII-hABCG2 cells. Data were normalized to the respective solvent control (0.1% DMSO) and set as 100% or 1 (mean ± SEM, N = 3, n ≥ 9, (A) and (B) one-way ANOVA with Holm-Šidák post hoc test, * significant difference in comparison to the control: *** $p \leq 0.001$, ** $p \leq 0.01$, * $p \leq 0.05$; (C) two-way ANOVA with Holm-Šidák post hoc test, # significant difference in comparison to MXN treatment alone: ### $p \leq 0.001$, ## $p \leq 0.01$, # $p \leq 0.05$).

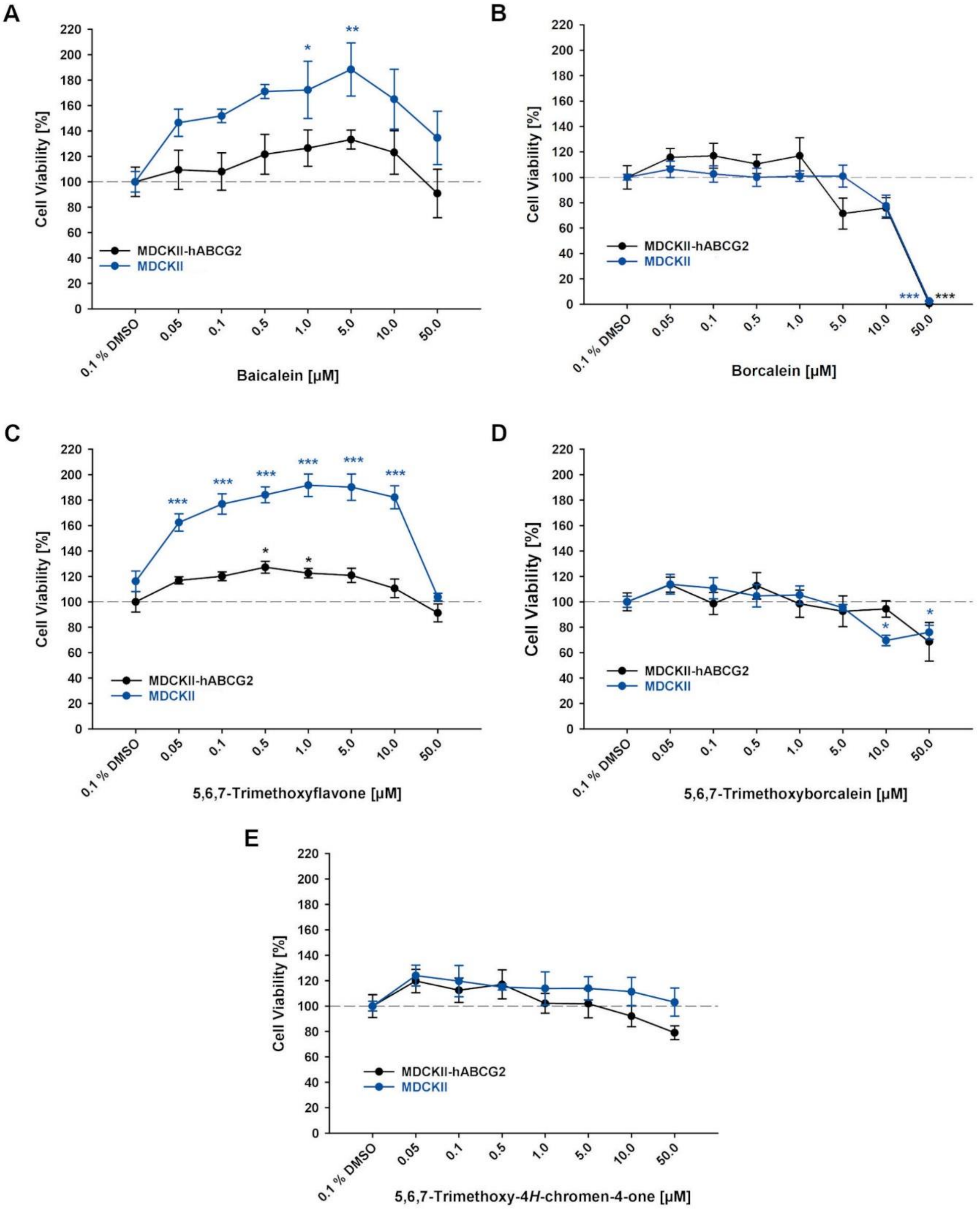

Fig. S2. Cytotoxicity of selected compounds. MDCKII cells were incubated with (A) baicalein, (B) borcalein, (C) 5,6,7-trimethoxyflavone, (D) 5,6,7-trimethoxyborcalein, and (E) 5,6,7-trimethoxy-4*H*-chromen-4-one in increasing concentrations for 48 h. Afterwards, cell viability was assessed by water-soluble tetrazolium 1 (WST-1) assay. Data were normalized to solvent control (0.1% DMSO) and set as 100% (mean ± SEM, N = 3, n ≥ 9, one-way ANOVA with Holm-Šidák post hoc test, * significant difference in comparison to the solvent control: *** $p \leq 0.001$, ** $p \leq 0.01$, * $p \leq 0.05$).

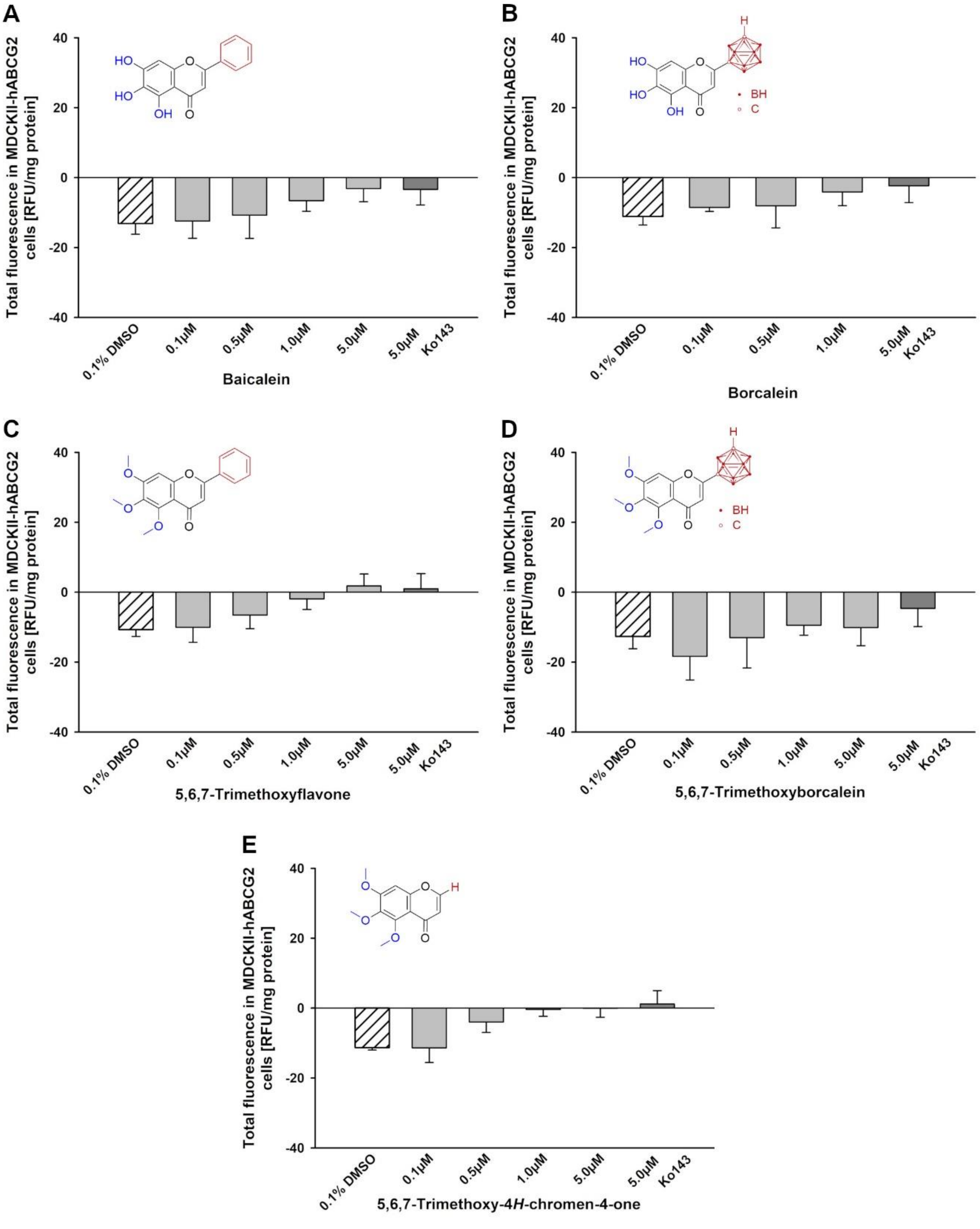

Fig. S3. Autofluorescence of selected compounds in MDCKII-hABCG2 cells. MDCKII-hABCG2 cells were incubated with (A) baicalein, (B) borcalein, (C) 5,6,7-trimethoxyflavone, (D) 5,6,7-trimethoxyborcalein, and (E) 5,6,7-trimethoxy-4*H*-chromen-4-one for 4 h and afterwards, cells were lysed and intracellular fluorescence was determined as described (mean ± SEM, N = 3, one-way ANOVA with Holm-Šidák post hoc test, * significant difference in comparison to the solvent control: $p \leq 0.05$).

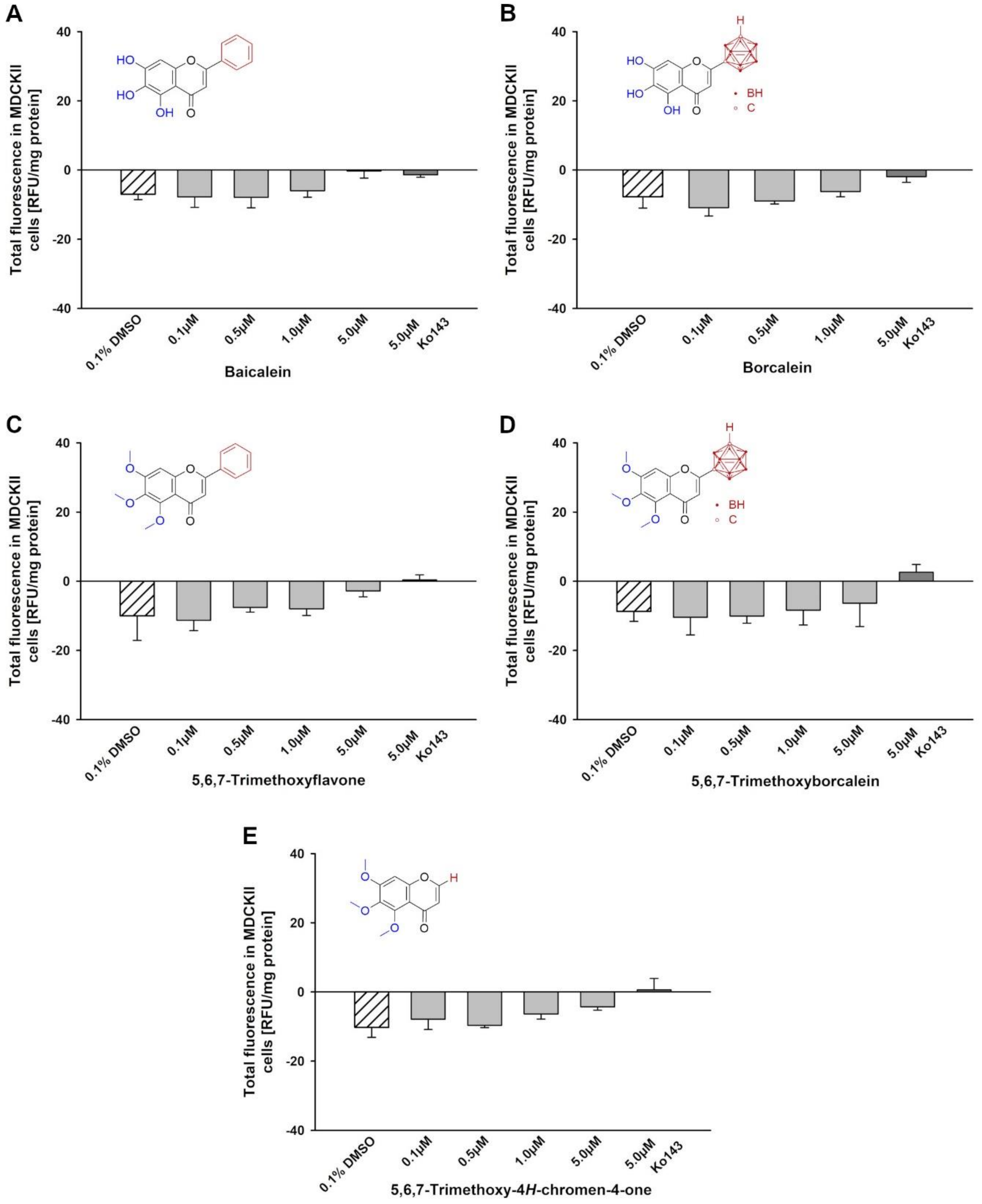

Fig. S4. Autofluorescence of selected compounds in MDCKII cells. MDCKII cells were incubated with (A) baicalein, (B) borcalein, (C) 5,6,7-trimethoxyflavone, (D) 5,6,7-trimethoxyborcalein, and (E) 5,6,7-trimethoxy-4*H*-chromen-4-one for 4 h and afterwards, cells were lysed and intracellular fluorescence was determined as described (mean ± SEM, N = 3, one-way ANOVA with Holm-Šidák post hoc test, * significant difference in comparison to the solvent control: $p \leq 0.05$).

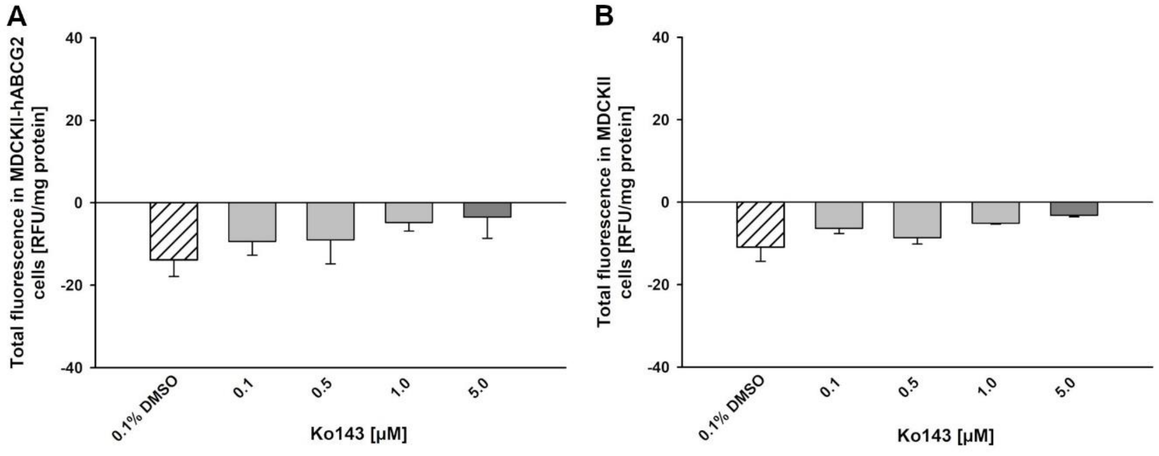

Fig. S5. Autofluorescence of Ko143. (A) MDCKII-hABCG2 and (B) MDCKII cells were incubated with Ko143 for 4 h and afterwards, cells were lysed and intracellular fluorescence was determined as described (mean ± SEM, N = 3, one-way ANOVA with Holm-Šidák post hoc test, * significant difference in comparison to the solvent control: $p \leq 0.05$).

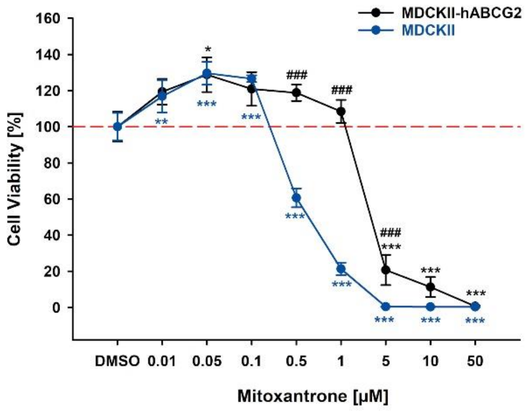

Fig S6. Cytotoxicity of mitoxantrone towards MDCKII-hABCG2 and MDCKII cells. MDCKII cells were treated with increasing concentrations of mitoxantrone for 48 h. Afterwards, cell viability was measured by WST-1 and an IC_{50} value of 2.600 ± 0.739 µM and 0.520 ± 0.075 µM was calculated for MDCKII-hABCG2 and MDCKII cells, respectively. Data are shown as mean SEM (N = 3, n = 9, *significantly different compared to solvent control determined by one-way-ANOVA with Holm-Šidák post hoc test: *** p ≤ 0.001, ** p ≤ 0.01, * p ≤ 0.05; # significant difference between MDCKII-hABCG2 and MDCKII cells determined by two-way ANOVA with Holm-Šidák post hoc test: ### p ≤ 0.001, ## p ≤ 0.01, # p ≤ 0.05).

2 Tables

Table S1. Crystal data of 5,6,7-trimethoxyborcalein.

Empirical formula	$C_{14}H_{22}B_{10}O_5$	
Formula weight	378.41	
Temperature	130(2) K	
Wavelength	71.073 pm	
Crystal system	Triclinic	
Space group	$P\overline{1}$	
Unit cell dimensions	a = 703.71(3) pm	α = 84.275(3)°
	b = 1059.38(5) pm	β = 83.074(3)°
	c = 1352.50(5) pm	γ = 75.978(4)°
Volume	0.96849(7) nm^3	
Z	2	
Density (calculated)	1.298 Mg/m^3	
Absorption coefficient	0.083 mm^{-1}	
F(000)	392	
Crystal size	0.4 x 0.3 x 0.1 mm^3	
Theta range for data collection	2.412 to 37.684°	
Index ranges	−12 ≤ h ≤ 11, −17 ≤ k ≤ 17, −22 ≤ l ≤ 23	
Reflections collected	27082	
Independent reflections	9799 [R(int) = 0.0307]	
Completeness to θ = 36.320°	100.0%	
Absorption correction	Semi-empirical from equivalents	
Max. and min. transmission	1 and 0.9656	
Refinement method	Full-matrix least-squares on F^2	
Data / restraints / parameters	9799 / 0 / 350	
Goodness-of-fit on F^2	1.034	
Final R indices [I > 2θ(I)]	R1 = 0.0482, wR2 = 0.1202	
R indices (all data)	R1 = 0.0739, wR2 = 0.1349	
Residual electron density	0.496 and −0.220 e·$Å^{-3}$	
CSD deposition Number	2221937	

Comments: Structure solution with SHELXT-2014 (dual-space method) [1]. Anisotropic refinement of all non-hydrogen atoms with SHELXL-2018 [2]. All H atoms were located on difference Fourier maps calculated at the final stage of the structure refinement.

Table S2. Binding free energies of selected compounds towards human ABCG2 transporter in docking simulations.

	**Binding free energy [kcal·mol^{-1}]**
**Baicalein**	–2.2
**Borcalein**	–2.0
**5,6,7-Trimethoxyflavone**	–5.7
**5,6,7-Trimethoxyborcalein**	–5.0
**5,6,7-Trimethoxy-4*H*-chromen-4-one**	–2.8

Table S3. Detected left shift factors. Left shift factors were calculated as IC_{50} MXN/IC_{50} MXN in combination with investigated compound determined in MDCKII-hABCG2 cells using WST-1 assay. IC_{50} values are given as mean ± SEM, N = 3, n ≥ 9, two-way ANOVA, * represents significant difference in comparison to a single MXN treatment, (*** $p < 0.001$).

**Treatment of MDCKII-hABCG2**	**IC_{50} [µM]**	**Left shift factor**	**Comparison to MXN**
**MXN**	2.649 ± 0.594		
**MXN + 1 µM Ko143**	0.295 ± 0.063	5.7-fold	***
**MXN + 5 µM Ko143**	0.177 ± 0.051	9-fold	***
**MXN + 5 µM Baicalein**	0.310 ± 0.073	5-fold	***
**MXN + 5 µM 5,6,7-Trimethoxyflavone**	0.693 ± 0.053	2.5-fold	No
**MXN + 1 µM 5,6,7-Trimethoxyborcalein**	0.222 ± 0.021	8.5-fold	***
**MXN + 5 µM 5,6,7-Trimethoxyborcalein**	0.091 ± 0.005	21.4-fold	***

SI References

1 Sheldrick GM (2015), SHELXT – Integrated space-group and crystal-structure determination. Acta Cryst. A (Acta Crystallographica Section A, Foundations and Advances) 71, 3–8.

2 Sheldrick GM (2015), Crystal structure refinement with SHELXL. Acta Cryst. C (Acta Crystallographica Section C, Structural Chemistry) 71, 3–8.
